# A novel selective cooling system for the brain: feasibility study in rabbits vs piglets

**DOI:** 10.1186/s40635-018-0211-4

**Published:** 2018-11-01

**Authors:** Mohammad Fazel Bakhsheshi, Lynn Keenliside, Ting-Yim Lee

**Affiliations:** 1 Imaging Program, Lawson Health Research Institute, London, Ontario Canada; 2 Imaging Research Laboratories, Robarts Research Institute, 100 Perth Drive, P.O. Box 5015, London, Ontario N6A 5k8 Canada; 3 Departments of Medical Imaging and Biophysics, The University of Western Ontario, London, Ontario Canada

**Keywords:** Brain temperature, Se brain cooling, Vortex tube, Rabbit, Nasopharyngeal brain cooling, Carotid rete

## Abstract

**Background:**

Selective brain cooling (SBC) methods could alleviate the complications associated with systemic hypothermia. The authors (MFB, LK, and T-YL) have developed a simple and an effective nasopharyngeal SBC method using a vortex tube. The primary focus of the study is to evaluate the effectiveness of this approach on rabbits and compare it with our previous published finding on piglets, which are mammals without and with a carotid rete, respectively.

**Methods:**

Experiments were conducted on six rabbits. Body temperature was measured continuously using an esophageal temperature probe while brain temperature was measured with an implanted thermometer. Two successive experiments were performed on each animal. In the first experiment, brain cooling was initiated by blowing room temperature air from the hospital medical air outlet, at a flow rate of 14–15 L/min into both nostrils for 60 min. The second series of measurements and brain cooling was performed in the same manner as the first one but blowing cold air (− 7 °C) at the same flow rate.

**Results:**

One hour post cooling with room temperature air at a flow rate of 14–15 L/min, the brain temperature was 34.2 ± 1.2 °C which resulted in mean brain cooling rates of 3.7 ± 0.9 °C/h. Brain temperature could be reduced more rapidly at mean rates of 5.2 ± 1.9 °C/h, while the body temperature as measured by the esophageal temperature probe was maintained above 36 °C during cooling and maintaining period.

**Conclusions:**

We have demonstrated that using the vortex tube allows initial rapid and SBC in rabbits. Moreover, comparing results between piglets and rabbits demonstrates clearly that the lack of a carotid rete does not prevent specific cooling of the brain by means of the nasopharyngeal method.

## Introduction

Therapeutic hypothermia has become an effective neuroprotective strategy as it can inhibit multiple pathways involved in ischemia/reperfusion injury [1–3]. Although some clinical studies indicate that the temperature range associated with better outcomes appears to be 32 to 35 °C [4, 5], a recent study revealed no significant difference between hypothermic and near-normothermic treatment groups (33 °C and 36 °C) in patients after cardiac arrest in terms of their survival and neurologic outcome [6]. Currently, most brain cooling methods rely on cooling down the whole body; however, decreasing the whole-body temperature below 34 °C can induce severe complications [7–9]. Therefore, selective rapid brain cooling and temperature control is beneficial to implement than whole-body cooling. To achieve this goal, different selective brain cooling (SBC) methods have been investigated to minimize the complications associated with systemic hypothermia by selectively cooling the brain. However, the limitations of the existing cooling technologies such as insufficient cooling and use of a relatively expensive coolant and/or irritant effects on skin contact points translate to their relative lack of effectiveness for the clinical conditions.

Cooling the nasal cavities lowers brain temperature due to the anatomic proximity of the internal carotid artery to the cavernous sinus [10]. Feasibility of intranasal evaporative cooling by RhinoChill device was successfully tested in patients after cardiac arrest [11, 12]. In the PRINCE trial, the RhinoChill was able to decrease tympanic (as a surrogate of the brain) temperature in patients to 34.2 °C in ~ 34 min [13]. Likewise, in a recent study [14], this has also been confirmed in stroke patients (− 1.2 °C after 58 min). The RhinoChill device vaporizes perfluorocarbon along with oxygen at a flow rate of 40–60 L/min with a catheter system into the nasal cavity leading to a fast induction of hypothermia. However, because prevention of brain injury may require up to 12–24 h of brain cooling, the cost for the coolant will be prohibitive if Rhinochill is used. Recently [15], we demonstrated a simple nasopharyngeal brain cooling method for lowering brain temperature based on blowing air into the nostrils at different temperatures and flow rates in a pig model [15]. However, mammals like pigs possess a carotid rete (a set of small arteries) which is a vast vascular network arising from the carotids at the base of the brain. The rete is surrounded by the cavernous sinus which receives cool blood from the nasal mucosa and face; together, these serve as an effective heat exchanger for the brain. In mammals with the carotid rete, arterial blood, on its way to the brain, is cooled in the carotid rete via heat exchange with cool venous blood returning from the nasal mucosa and face [16]. In mammals in which the carotid rete is missing (like in humans), some have suggested that there is no effective heat exchange in the cavernous sinus and, consequently, SBC is not efficient in these species [16]. Yet, several mammals, including horses, rabbits, and rats, lack a carotid rete but clearly demonstrate SBC [17–19]. To the best of our knowledge, there have been no previous studies comparing the efficiency of nasopharyngeal brain cooling on pigs and rabbits, which are mammals with and without a carotid rete, respectively.

The present study was an incremental work based on our previous research and was designed to evaluate the effectiveness of this approach on rabbits and compare it with our previously published findings on piglets [20, 21]. Maintenance of the brain-body temperature gradient following cooling was also explored.

## Materials and methods

### Animal preparation and experimental procedure

Experiments were conducted on six male New Zealand white rabbits, approximately 6 months old (weight = 3.5 ± 0.2 kg). All animal experiments were approved by the Animal Use Subcommittee of the Canadian Council of Animal Care at Western University. Animals were induced and maintained with isoflurane gas anesthesia at 4% and 2–3% concentrations, respectively. The 4% isoflurane provided a rapid induction of anesthesia within 15–30 s while the 2–3% isoflurane maintained surgical anesthesia, allowing surgical procedures to be performed without any physiological signs of pain or changes of hemodynamic parameters. The animal was intubated with a cuffed endotracheal tube and ventilated with a volume-controlled mechanical ventilator to deliver oxygen/medical air mixture (2:1). Body temperature was measured continuously using an esophageal temperature probe attached to a Surgivet monitor (Temperature Probe WWV3418, Smiths Medical, Dublin, OH, USA). A 1–2-mm burr hole was drilled in the skull 1.5 posterior and 1.5 lateral to the bregma along the mid-line with a Dremel tool. The needle thermocouple probe (Digi-Sense, Type-K, Needle Microprobe, Mini Conn 0.75"L .020 Dia, GRD 5Ft FEP Cable) was inserted through the burr hole into the brain to a depth of ≈ 2 cm from the brain surface to measure brain temperature. A femoral artery was catheterized to monitor heart rate (HR) and mean arterial blood pressure (MAP) and to intermittently collect arterial blood samples for gas and electrolyte levels (*p*_*a*_CO_2_, *p*_*a*_O_2_, *S*_*a*_O_2_, *cNa*^*+*^, *cK*^*+*^, *cCl*^*−*^, and *cCa*^*2+*^) and pH analyses. Arterial blood was drawn every 30 min to measure arterial blood gas with an analyzer (ABL80 FLEX CO-OX, Radiometer Medical ApS, DK-2700, Brønshøj, Denmark).

After surgery, each animal together with a recirculating hot water pad was wrapped with linen blankets, maintained on 2–3% isoflurane, and 30–40 min were allowed for baseline physiological parameters to stabilize before the nasopharyngeal brain cooling was started. Two successive experiments were performed on each animal. In the first experiment, nasopharyngeal brain cooling was initiated by blowing room temperature air (21 °C ± 1 °C and relative humidity (RH) of 15–20%), delivered from a hospital medical air outlet, at a flow rate of 14–15 L/min as measured by a flow meter (VWR Flow Meters Acrylic, FR4500 series with accuracy of ± 3%, VWR International Inc) into both nostrils for 60 min (group I). The brain was then allowed to gradually rewarm to baseline temperature by turning off the hospital air supply. In the second experiment, brain cooling was accomplished by blowing cold air (− 7 °C, RH 15–20%) at the same flow rate as the first experiment for 60 min. Once the brain temperature stabilized, the flow rate and air temperature were adjusted to maintain the brain temperature and reach for another 60 min while core body temperature was maintained above 36 °C using the recirculating hot water pad and by packing gloves filled with hot water around the body (group II). Cold air was generated by the vortex tube, as discussed in detail in the following section. During cooling, the mouth of the animal was kept open so that air can escape from mouth and nostrils.

Arterial CO_2_ tension (*p*_*a*_CO_2_) was monitored throughout the experiments, either directly by blood gas measurements or by the end-tidal CO_2_ tension (EtCO_2_), and maintained at normocapnia between 37 and 42 mmHg by adjusting the breathing rate and volume. EtCO_2_, tidal volume, respiratory rate, pulse oximetry (SpO_2_), and heart rate were continuously measured using a multi-parameter monitor (Surgivet Advisor Vital Signs Monitor V9200, Smiths Medical, Dublin, OH, USA). Each experiment was completed in 8–9 h, and the animal was sacrificed with intravenous potassium chloride (1–2 ml/kg, 2 mEq/mL) infusion at the end of the experiments.

The experimental procedures used on piglets were similar to those used on rabbits [20, 22]. These data are presented from our previous study in piglets [21]. Briefly, experiments were conducted on 12 piglets (7 females and 5 males) with an average age of 44 ± 9 h and an average weight of 2.9 ± 1.3 kg. Following surgery, each piglet was wrapped with a linen blanket that was used to maintain the piglets normothermic at 38 ± 0.5 °C for 60 min prior to the initiation of cooling. The temperature control was then discontinued, and the piglets were randomized to the following nasopharyngeal brain cooling treatments: room temperature at a flow rate of 14–15 L/min (*n* = 6) and − 7 °C at a flow rate of 14–15 L/min (*n* = 6). In all of the experimental studies, anesthesia was maintained with 1–2% isoflurane until the end of the experiment. Cold air was generated by circulating air, delivered from a pressurized tank, through a custom-made heat exchanger [20, 22].

### Method of nasopharyngeal brain cooling

**Fig. 1:**
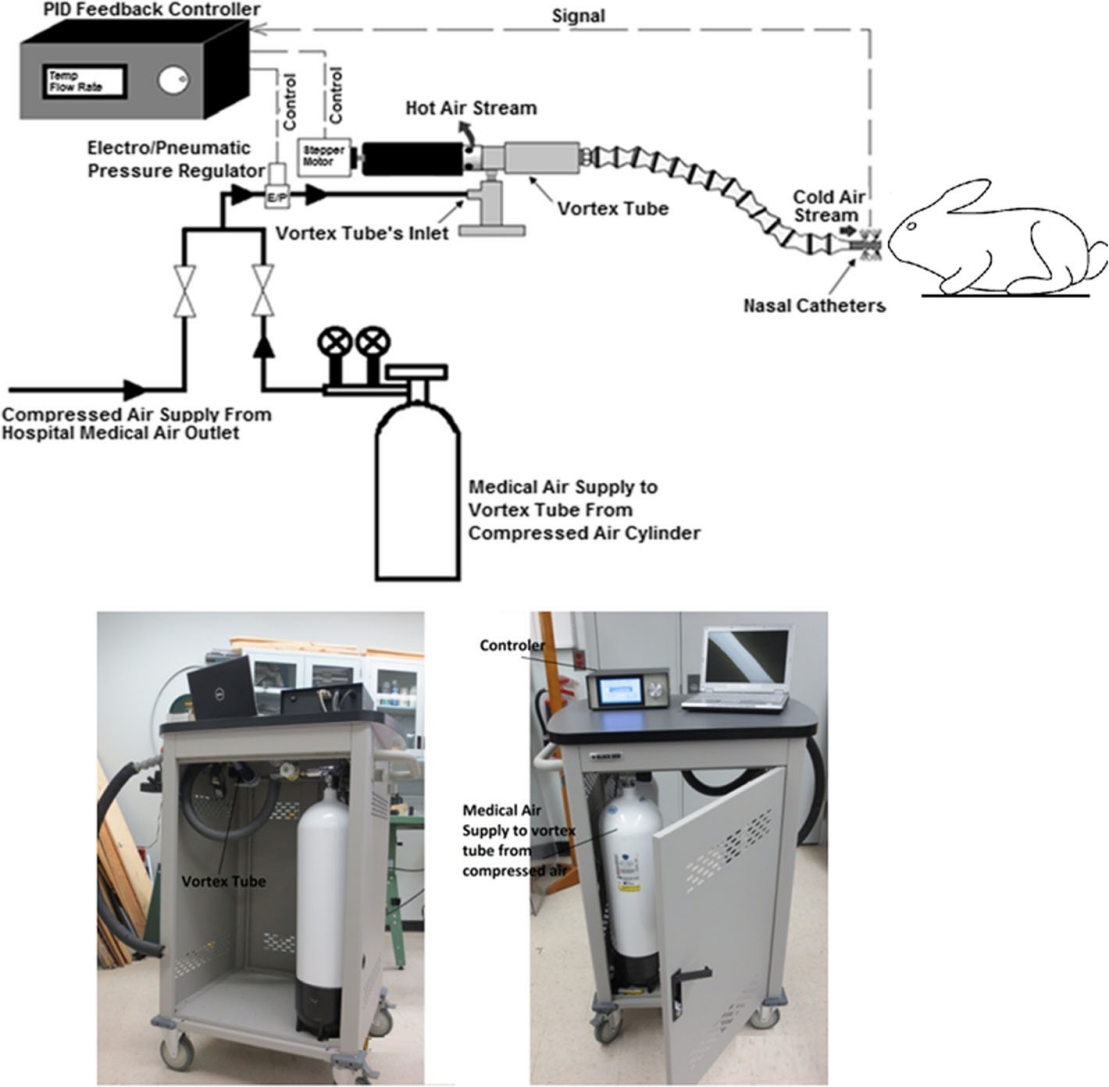
Schematic representation of the cooling circuit used for nasopharyngeal brain cooling

The vortex tube (adjustable cold air gun, ITW Vortec Ltd), shown in Fig. 1, is a mechanical device that is used to generate cold air without any moving parts, chemical reactions, or external energy supply. Since the vortex tube contains no other parts inside the tube, the generation of an air streams at different temperature can only be attributed by the effects of fluid dynamics. Different hypotheses have been proposed for the basis of temperature separation. Nevertheless, a well-accepted explanation for the thermal phenomenon within the vortex tube has not been proposed due to the complex internal flow mechanism. Recently, an explanation was reported that the separation of energy within a vortex tube was achieved by the turbulent eddies, which carry the heat from the core to the periphery [23]. However, the similarity between this explanation and the secondary circulation [24] and the lack of new evidence indicate the unclear mechanism within a vortex tube. In our study, a source of compressed medical air (supplied either by L’Air Liquide Ltd. in cylinders of capacity of 232 ft^3^ at fill-pressure of 2265 PSI downregulated to 50 PSI or from a hospital medical outlet at a fixed outlet pressure of 50 PSI) is applied to the inlet nozzle. Air passes through the generation chamber of the vortex tube which creates the vortex inside the tube and separates the compressed air stream into cold and hot streams. The fraction of compressed air exiting as cold air (also referred as cold fraction) was adjusted by a throttle needle valve. aBoth the temperature and flow rate of the cold air stream are controlled and monitored continuously. The temperature at the cold air outlet was monitored and recorded continuously with a thermometer (Thermometer/Data Logger, HH309A, with Four Type K Thermocouple Inputs, Omega Engineering, Stamford CT; resolution 0.1 °C). A thermistor was also placed inside one of the two nasal catheters to monitor the temperature of the cold air inside the nasal cavity throughout the experiments. Figure 1 shows the schematic of the experimental setup. Nasopharyngeal brain cooling was achieved by connecting the two nasal catheters (made from polyvinyl chloride, PVC) to the cold air outlet of a vortex tube via tubing. The nasal catheters were coated with 2% lidocaine gel for local anesthesia during insertion and were inserted 4–5 cm into each nostril***.***

### Statistical analysis

SPSS 17.0.0 (SPSS, Inc., Chicago, IL) was used for all statistical analyses. Comparisons of vital signs were analyzed by two-way analysis of variance (ANOVA) with group and time as the two factors followed by post hoc test with Bonferroni correction to determine statistical differences at different times within a group and between groups at different times. Statistical significance was declared when *p* value was < 0.05. All numeric data are presented as mean ± standard deviation (SD) unless otherwise noted.

## Results

Table 1 displays a summary of the measured physiological parameters (*S*_*a*_O_2_, *c*Na^+^, *c*K^+^, *c*Cl^−^, *c*Ca^2+^, MAP, HR, pH, *P*_*a*_CO_2,_ and *t*Hb) in the two groups, prior to and during cooling. There was no significant difference in any physiological parameter between the groups at baseline. In group I, 1 h after the initiation of cooling, when the mean brain temperature dropped to 34.5 ± 0.9 °C, a statistically significant (*p* < 0.05) decrease in HR and MAP were observed, but *c*K^+^ started increasing after initiation of cooling. Similarly, in group II, there were statistically significant decreases in HR and MAP when the brain temperature dropped to 35.4 ± 0.9 °C and 33.2 ± 1.2 °C after 30 min and 1 h of cooling, respectively, and remained significantly depressed (relative to baseline) for the duration of the study. As well, a statistically significant increase in *c*K^+^ was observed after 2 h of cooling. No arrhythmias were noted during cooling or rewarming. No incidences of catheter thrombosis, acute infection, or other complications associated with the insertion procedure were observed.

**Table 1:** Physiological parameters measured at different times during SBC within groups in rabbits

	Baseline	Cooling		
	1–60 min	30 min	1 h	2 h
Brain temperature (°C)				
Group I: 14–15 L/min at RT	38.2 ± 0.7	35.2 ± 0.7*	34.5 ± 0.9*	N/A
Group II: 14–15 L/min at − 7 °C	37.8 ± 0.4	35.4 ± 0.9*	33.2 ± 1.2*	32.1 ± 1.7*
S_a_O_2_ (%)				
Group I: 14–15 L/min at RT	100	100	100	N/A
Group II: 14–15 L/min at − 7 °C	100	100	100	100.0
cNa^+^ (mmol/L)				
Group I: 14–15 L/min at RT	141 ± 3	142 ± 2	142 ± 3	N/A
Group II: 14–15 L/min at − 7 °C	144 ± 2	142 ± 1	141 ± 1	141 ± 2
cK^+^ (mmol/L)				
Group I: 14–15 L/min at RT	3.4 ± 0.3	3.5 ± 0.2	3.7 ± 0.1	N/A
Group II: 14–15 L/min at − 7 °C	4.4 ± 0.2	4.5 ± 0.5	4.6 ± 0.3	5.1 ± 0.3*
cCa^2+^ (mmol/L)				
Group I: 14–15 L/min at RT	1.2 ± 0.1	1.0 ± 0.1	1.3 ± 0.1	N/A
Group II: 14–15 L/min at − 7 °C	1.1 ± 0.1	1.3 ± 0.1	1.2 ± 0.1	1.2 ± 0.1
cCl ^−^ (mmol/L)				
Group I: 14–15 L/min at RT	102 ± 2	104 ± 2	102 ± 2	N/A
Group II: 14–15 L/min at − 7 °C	110 ± 3	104 ± 3	103 ± 1	108 ± 2
MAP (mmHg)				
Group I: 14–15 L/min at RT	37 ± 6	34 ± 6	30 ± 5*	N/A
Group II: 14–15 L/min at − 7 °C	35 ± 4	32 ± 7	27 ± 6*	25 ± 6*
HR (bpm)				
Group I: 14–15 L/min at RT	272 ± 20	250 ± 14*	226 ± 16*	N/A
Group II: 14–15 L/min at − 7 °C	275 ± 11	247 ± 18*	215 ± 20*	204 ± 15*
pH				
Group I: 14–15 L/min at RT	7.4 ± 0.1	7.4 ± 0.1	7.4 ± 0.1	N/A
Group II: 14–15 L/min at − 7 °C	7.4 ± 0.1	7.3 ± 0.1	7.3 ± 0.1	7.3 ± 0.1
tHb (g/dL)				
Group I: 14–15 L/min at RT	11.1 ± 1.0	12.2 ± 0.8	12.5 ± 1.0	N/A
Group II: 14–15 L/min at − 7 °C	12.4 ± 1.0	12.1 ± 0.3	12.4 ± 0.9	12.6 ± 0.7
paCO_2_ (mmHg)				
Group I: 14–15 L/min at RT	38 ± 2	40 ± 2	37 ± 2	N/A
Group II: 14–15 L/min at − 7 °C	37 ± 2	39 ± 3	40 ± 1	41 ± 2

*SaO*_*2*_ oxygen saturation, *MAP* mean arterial blood pressure, *HR* heart rate, *tHb* total hemoglobin in blood, *P*_*a*_*CO*_*2*_ arterial oxygen partial pressure, *cNa*^*+*^ sodium concentration, *cK*^*+*^ potassium concentration, *cCa*^*2+*^ calcium concentration, *cCl*^*−*^ chloride concentration, *RT* room temperature (21 °C ± 1 °C), *N/A* not available

*A statistically significant (*p* < 0.05) difference compared to the baseline

**Fig. 2:**
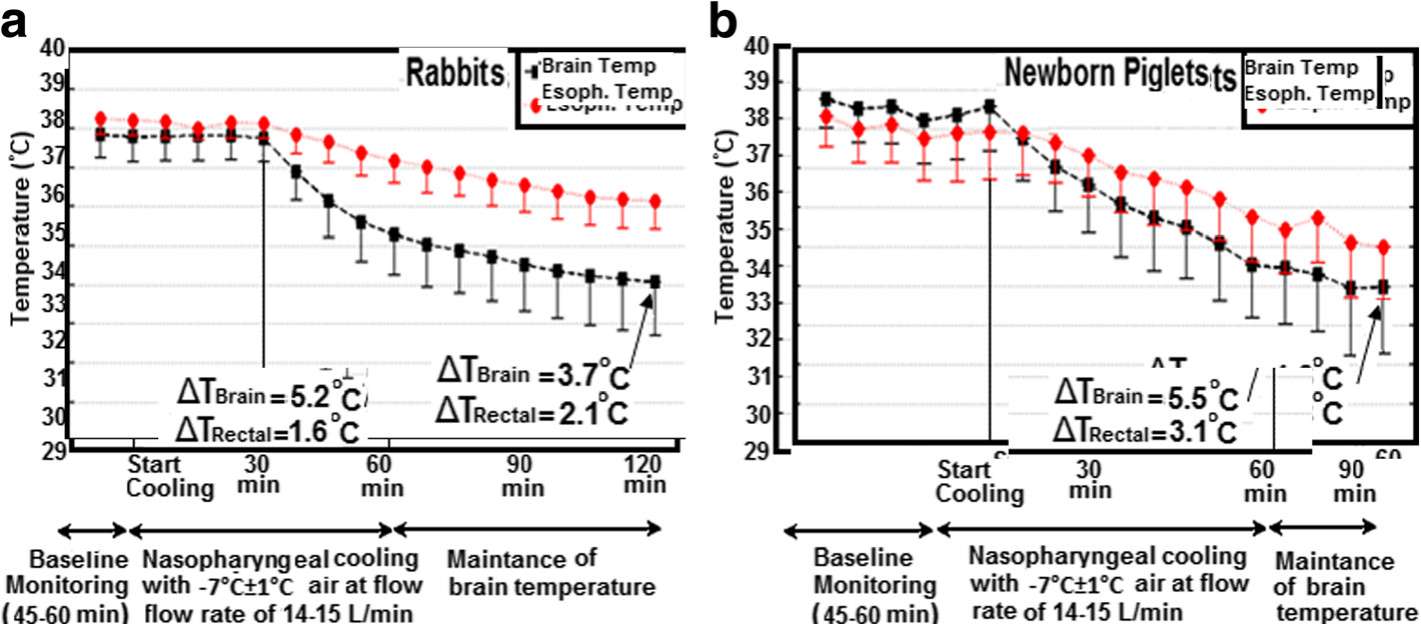
Brain and esophageal temperature over time for nasopharyngeal cooling method with room temperature air at a flow rate of 14–15 L/min on **a** rabbits (*N* = 6) and **b** piglets (*N* = 6, all piglet data were taken from previous work of our group [21])

Changes in esophageal and brain temperatures in rabbits are shown in Fig. 2a. During the baseline monitoring period of 40–60 min, both esophageal and brain temperature did not vary more than 0.1 ± 0.1 °C from the baseline. With the initiation of nasopharyngeal cooling with room temperature air at a flow rate of 14–15 L/min, the brain temperature decreased from 37.9 ± 0.5 °C to 35.6 ± 0.9 °C within 15 min. One hour post cooling, the brain temperature reached 34.2 ± 1.2 °C which resulted in a mean brain cooling rate of 3.7 ± 0.9 °C/h, as displayed in Fig. 2a. The esophageal temperature decreased during the same interval from 38.3 ± 0.3 °C to 36.2 ± 0.6 °C which corresponded to a cooling rate of 2.1 ± 0.4 °C/h. Figure 2b shows the brain and esophageal temperature as a function of time of piglets which underwent nasopharyngeal brain cooling in the same manner as the rabbits. Both brain and esophageal temperature decreased from 38.4 ± 0.7 °C and 38.2 ± 0.7 °C to 33.9 ± 1.7 °C and 35.4 ± 1.7 °C. These changes in brain and esophageal temperatures corresponded to cooling rates of 4.5 ± 1.2 °C/h and 2.8 ± 0.6 °C/h, respectively, as displayed in Fig. 2b.

**Fig. 3:**
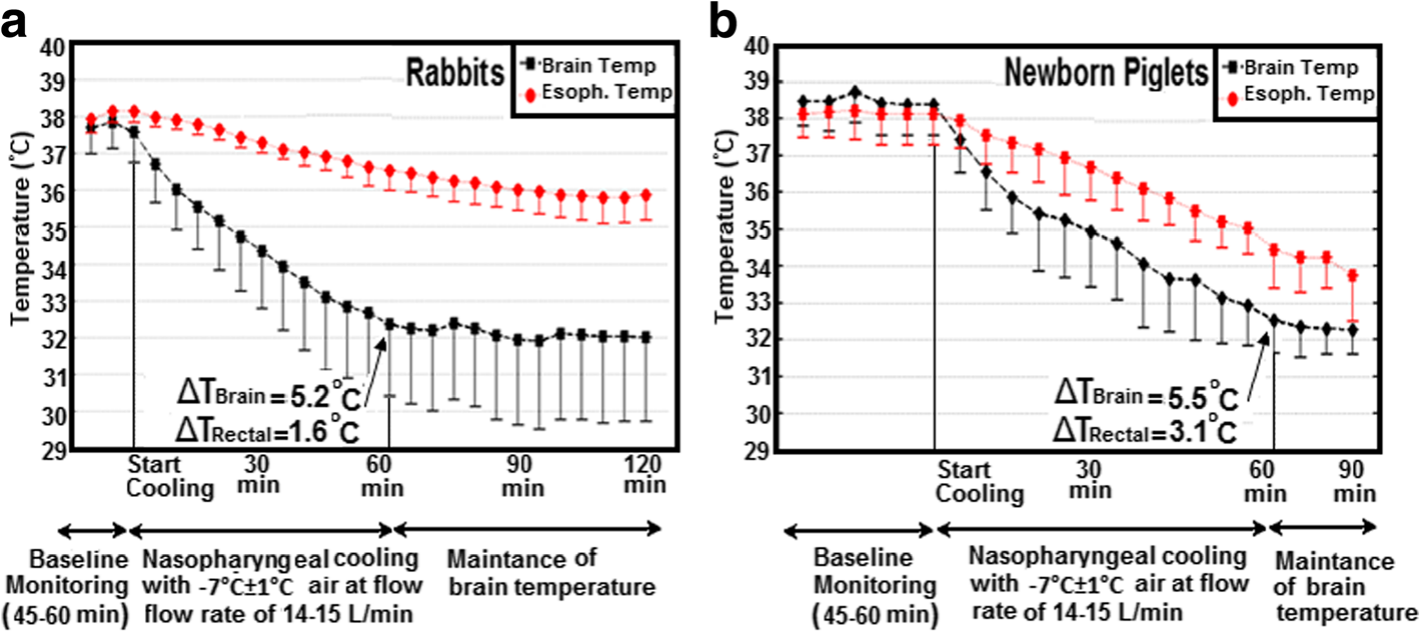
Brain and esophageal temperature over time for nasopharyngeal cooling method with cold air at a flow rate of 14–15 L/min on **a** rabbits (*N* = 6) and **b** piglets (*N* = 6, all piglet data were taken from previous work of our group [21])

Following 60 min of cooling with room temperature air at a flow rate of 14–15 L/min, the air flow was stopped, and rewarming was initiated with the use of recirculating hot water pad and by packing gloves filled with hot water around the body of the animal. The return to the baseline pre-cooling temperature was usually achieved in 80 ± 15 min. During this phase, the brain and esophageal temperatures increased by 2.9 ± 0.7 °C/h and 1.7 ± 0.5 °C/h, respectively. Temperatures in the rewarming period between the cooling episodes were not shown in Figs. 2 and 3.

Figure 3a shows that with the use of − 7 °C instead of room temperature air at the same flow rate, a faster cooling rate was achieved in rabbits. Mean brain and esophageal temperatures decreased to 32.3 ± 2.1 °C and 36.5 ± 0.5 °C within 60 min of cooling from baseline temperature of 37.5 ± 0.8 °C and 38.1 ± 0.3 °C, which corresponded to cooling rates of 5.2 ± 1.9 °C/h and 1.6 ± 0.4 °C/h, respectively. The brain-body temperature gradient, calculated as the difference between brain and esophageal temperature, peaked about 55 min after the initiation of the cooling. This gradient could be maintained within ± 0.5 °C for another hour by increasing the air temperature to 4 ± 4 °C and adjusting the air flow rate to 20 ± 10 L/min. Similarly, in piglets, Fig. 3b shows that brain and esophageal temperatures were reduced more rapidly at a rate of 5.5 ± 1.1 °C/h and 3.1 ± 1.1 °C/h by using − 7 °C instead of room temperature air at the same flow rate.

**Fig. 4:**
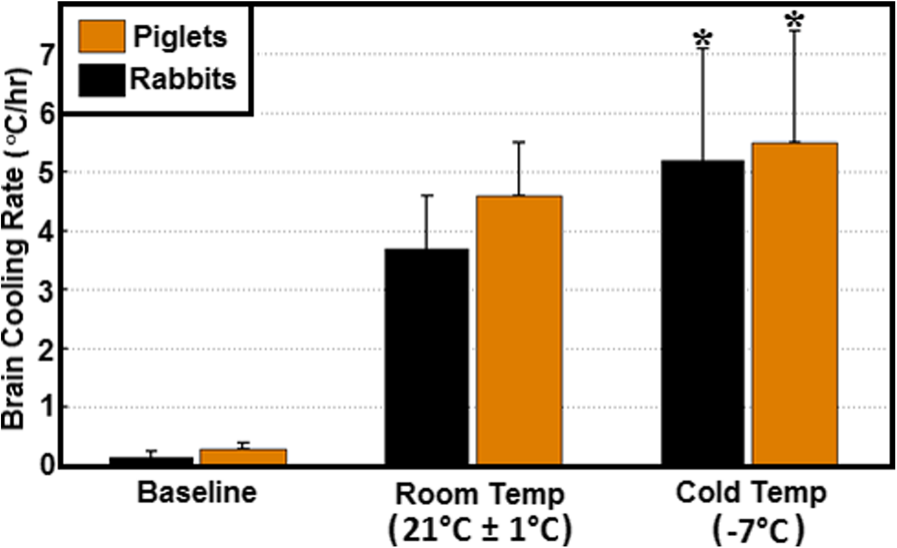
Mean brain cooling rate with different nasopharyngeal cooling methods on rabbits and piglets. Asterisk signifies a statistically significant (*p* < 0.05) difference between cold temperature versus room temperature in the same species

Figure 4 shows the average brain cooling rates achieved using the nasopharyngeal brain cooling method with either room temperature or cold air at a flow rate of 14–15 L/min for rabbits and piglets. Brain cooling rate was significantly greater with cold than room temperature air in both species. The brain cooling rates achieved in rabbits and piglets using the nasopharyngeal cooling method with either cold or room temperature air were not significantly different.

## Discussion

The presented study has demonstrated that blowing cold air produced by the vortex tube into nasal cavities is an effective method to specifically reduce brain temperature and maintain it at such level in rabbits. In both animal species, nasopharyngeal brain cooling was accomplished by blowing room or cold air temperature into the nostrils at a flow rate of 14–15 L/min. The only difference in the source of cold air is as follows: for the rabbit experiments, the cold air was produced with a vortex tube whereas for the piglet experiments, cold air was from an in-house heat exchanger. In rabbits, brain cooling at the rate of 3.7 ± 0.9 °C/h was achieved by setting the flow rate of room temperature air to 14–15 L/min. Similarly, in piglets, with the initiation of nasopharyngeal cooling with the same setting, the brain temperature decreased to 33.9 ± 1.7 °C from baseline (38.4 ± 0.7 °C) which resulted in mean brain cooling rates of 4.5 ± 1.2 °C/h. The rabbits and piglets had an average body temperature of 37.5 ± 0.8 °C and 38.2 ± 0.6 °C (range 37.5–38.8 °C), which was within the normal temperature range, respectively [25].

In the second series of experiments, we showed that SBC can be achieved more rapidly by blowing cold air (≈ − 7 °C) at a flow rate of 14–15 L/min into the nasal cavity; brain temperature in piglets and rabbits were reduced at mean rates of 5.2 ± 1.9 °C/h and 5.5 ± 0.9 °C/h, respectively. In rabbits, the maximum brain-esophageal temperature gradient of − 4.5 °C was reached about 50 min after the initiation of cooling and remained unchanged during the rest of nasopharyngeal cooling. Systemic adverse effects of hypothermia in general appear to be proportional to the degree of cooling [7]. Therefore, in order to minimize the complications associated with systemic hypothermia, the core body temperature was maintained above 36 °C, which is within the normal temperature range in humans [26]. However, in piglets, the core temperature continued to drop till it reached the cooled brain temperature. The most likely explanation for this discrepancy is that in rabbits once the brain temperature stabilized after 60 min of nasopharyngeal cooling, the core temperature was maintained > 36 °C using recirculating heated water blankets and hot water gloves; however in piglets, their body was only covered with linen sheets and no external heating sources were utilized.

A temperature of ~ − 7 °C and a flow rate of 14–15 L/min were chosen from the results of a small series of titration experiments in six pigs in which both temperature and flow rate were adjusted with the target of cooling the brain to 32–34 °C within 30–45 min. Although it is unlikely that air at subzero temperature will induce freezing damage to the mucosa and embedded blood vessels and nerves of the nasal cavity, we monitored the temperature inside the nasal cavity to avoid subzero temperature. A thermistor was placed at the tip of one of the two nasal catheters to measure the temperature inside the nasal cavity throughout the experiments. Even at a flow rate of 14–15 L/min and a temperature of ≈ − 7 °C, the air was warmed rapidly along the nasal catheters before reaching the nasopharyngeal tissue. An average temperature of about 6–7 °C was consistently measured with the catheter tip thermistor. In our previous study [27], we investigated whether there was any damage to the upper respiratory tract following ~ 7 h cooling in three pigs, and as a result, no nasal or nasopharynx mucosal swelling, necrosis, or hemorrhages were revealed on MRI images. However, more detail histopathology examination may be necessary in future studies.

Many studies have been reported that hypothermia can induce metabolic disturbances and lower electrolyte levels during cooling, such as hypokalemia (serum potassium levels less than 3.5 mmol/L) by a transcellular shift of potassium into the intracellular compartment [28, 29]. Such electrolyte disorders can increase the risk of arrhythmias and other potentially harmful complications [30, 31]. Therefore, frequent measurement of electrolytes during cooling is necessary to guide the appropriate amount of replenishment due to the risk of rebound hyperkalemia, as potassium can move back out of the cells when the patient is rewarmed. However, contrary to hypokalemia during cooling, a gradual increase in serum potassium level (i.e., hyperkalemia) was observed in this study, which could be due to the anesthetic effects [32, 33]. Therefore, it might be worth trying different anesthetic induction agents in future studies. Moreover, nasopharyngeal cooling in both groups was initially associated with peripheral vasoconstriction, tachycardia, and increased cardiac output. However, a progressive decrease in temperature led to bradycardia, decreased cardiac output, and hypotension which are likely a combined effect of temperature reduction and general anesthesia [34].

There are some limitations/concerns that need to be addressed prior to clinical situations. First, there are several anatomical differences between these animal species and humans (e.g., cerebral blood flow, distance from the nasopharynx to the brain, size of the brain, and the area in which heat exchange is carried out). Piglets and rabbits differ considerably to humans or larger animals in their ratio between the size of their body weight and their brain. As such, the effectiveness of this approach on brain cooling might not be the same on larger mammals. Second, the infusion rate at which brain cooling was achieved (i.e., 15 L/min) may be different in humans. Third, in all of the experimental studies, anesthesia was maintained with 1–2% isoflurane until the end of the experiment without any remarkable changes in blood pressure, heart rate, or oxygen saturation. Isoflurane was used as the main anesthetic in the present study, as its administration is easy to apply and quick to control the level of anesthesia. This avoids problems associated with injectable anesthetics, such as a lack of agents to reverse their activity rapidly in case of overdose or possible side-effects. Isoflurane has been shown to influence systemic arterial blood pressure and cardiac output only to a minimal degree over several hours of induction [35]. Experiments in animals have shown that isoflurane has effects on cerebral blood flow (CBF), cerebral metabolic rate for oxygen (CMRO_2_), and intracranial pressure (ICP). Isoflurane reduces CMRO_2_ and causes a minimum to moderate increase in CBF and ICP [36, 37] which have limited its use as a sedative agent in the neurosurgical intensive care unit. In our group, we showed that a typical anesthetic induction dose of propofol reduces blood pressure by ∼ 30% when the anesthetic was switched from isoflurane to propofol [38]. A final concern with this study is that the brain temperature was measured at one position and therefore, no information was delivered about the homogeneity of regional brain temperature. However, in two experiments, the temperature gradient within the rabbit’s brain, calculated as the difference between frontal and parietal lobes, was measured and was not more than 0.1 °C. The only disadvantage experienced with the use of the vortex tube during experiments was the continuous noise of escaping air, which can be minimized by installing all components in a mobile enclosed cart, which is commercially available.

## Conclusion

We have demonstrated that using the vortex tube allows initial rapid and specific brain cooling in rabbits. Moreover, comparing results between piglets and rabbits demonstrates that the lack of a carotid rete does not prevent specific cooling of the brain by means of the nasopharyngeal method. To evaluate the efficiency of the method and reproducibility of the cooling, we will switch to a large animal model, juvenile pigs, in our next set of experiments and explore maintenance of the brain-body temperature gradient for 6–7 h cooling and gradual rewarming rate (0.25 °C/h). In future studies, we will be blowing humidified air into nasal cavities and humidity will be measured and controlled inside of the nasal catheter right before nasal cavities. Histopathological studies on the lining of the nasal cavity will also be performed to demonstrate that there is no damage induced by blowing air for an extended period into the nostrils.
